# Inherent versus induced protein flexibility: Comparisons within and between apo and holo structures

**DOI:** 10.1371/journal.pcbi.1006705

**Published:** 2019-01-30

**Authors:** Jordan J. Clark, Mark L. Benson, Richard D. Smith, Heather A. Carlson

**Affiliations:** 1 Department of Medicinal Chemistry, University of Michigan, Ann Arbor, Michigan, United States of America; 2 Department of Computational Medicine and Bioinformatics, University of Michigan, Ann Arbor, Michigan, United States of America; University of Oxford, UNITED KINGDOM

## Abstract

Understanding how ligand binding influences protein flexibility is important, especially in rational drug design. Protein flexibility upon ligand binding is analyzed herein using 305 proteins with 2369 crystal structures with ligands (holo) and 1679 without (apo). Each protein has at least two apo and two holo structures for analysis. The inherent variation in structures with and without ligands is first established as a baseline. This baseline is then compared to the change in conformation in going from the apo to holo states to probe induced flexibility. The inherent backbone flexibility across the apo structures is roughly the same as the variation across holo structures. The induced backbone flexibility across apo-holo pairs is larger than that of the apo or holo states, but the increase in RMSD is less than 0.5 Å. Analysis of χ_1_ angles revealed a distinctly different pattern with significant influences seen for ligand binding on side-chain conformations in the binding site. Within the apo and holo states themselves, the variation of the χ_1_ angles is the same. However, the data combining both apo and holo states show significant displacements. Upon ligand binding, χ_1_ angles are frequently pushed to new orientations outside the range seen in the apo states. Influences on binding-site variation could not be easily attributed to features such as ligand size or x-ray structure resolution. By combining these findings, we find that most binding site flexibility is compatible with the common practice in flexible docking, where backbones are kept rigid and side chains are allowed some degree of flexibility.

## Author summary

Here, we examine how ligand binding affects protein flexibility by analyzing over 4000 crystal structures, an order of magnitude more than previous studies based on apo-holo pairs. A debate exists in the literature over how flexible binding sites are in proteins. Studies that conclude there is little motion upon ligand binding tend to measure backbone RMSD, but studies that show larger conformational change base their analyses on side-chain orientations. None of these studies have used the same proteins, so it is unclear how much the different conclusions are due to the chosen analyses versus the different datasets used. Furthermore, many studies have used apo-holo pairs to measure conformational change in proteins, but none have examined the inherent flexibility across the apo and holo states themselves. The induced change seen in an apo-holo pair must be placed in context of how variable the apo and holo states are. Our analyses reconcile any existing debate by confirming inherently different behavior for backbones and side chains, where backbones tend to sample very little conformational space and side chains are frequently pushed into new conformations upon ligand binding.

## Introduction

Proteins are naturally flexible biopolymers composed of a string of amino acids folded into a largely non-covalent structure.[1] The degree of flexibility is often tightly coupled to the protein’s function, especially for enzymes. Understanding the flexibility in proteins is important in protein folding, protein engineering, and rational drug design.

A key feature of protein-ligand binding sites is that they have both characteristically rigid and flexible residues.[2, 3] Rigidity can aid in specificity and tightness of ligand binding, while flexibility allows for entry of ligands into the binding site and can also be involved in communication between allosteric and orthosteric binding sites. Clusters of residues near binding sites are often observed in strained conformations.[4, 5] Ligand binding was seen to induce strain in these residues, and it was hypothesized that this increase in internal energy could be used by the protein for catalysis and ejecting a ligand from an active site.

Being able to fully account for induced changes is especially important in protein-ligand docking. Docking proves to be very difficult in practice when conformational changes occur upon binding.[6, 7] The cross-docking problem is illustrative of the difficulties of accounting for protein flexibility in ligand binding. Cross docking attempts to dock a ligand from one crystal structure into the binding site of another structure of the same protein, but research shows that many ligands do not fit unless the protein is allowed to adjust to the ligand.[8–11] The larger the required adjustment, the harder it is to accurately predict protein-ligand binding.[12] Protein flexibility needs to be incorporated to accurately represent protein-ligand binding.

As we outline below, there have been many studies examining the extent and properties of ligand binding by comparing apo and holo protein crystal structures. A number of studies have also examined the local characteristics of their binding sites, such as side-chain flexibility or solvent accessible surface area (SASA), while some studies have examined only global protein changes upon ligand binding. Analyses of most studies fell into two categories: root mean square deviation (RMSD) calculations of backbone atoms or rotameric analysis of amino acid side chains. These different approaches have led to conflicting conclusions which our study helps to reconcile. Below, we summarize the most significant findings to date.

### Backbone analysis

Structural variation appears small when assessed through backbone motion. Gutteridge and Thornton found that enzymes in their small dataset of 11 proteins (11 apo, 14 holo) bound to either a substrate or product tended to be more structurally similar to each other than to free enzyme (substrate-bound and product-bound structures had an average C_α_ RMSD of 0.36 Å while apo enzymes averaged 0.75 Å RMSD to the substrate structures and 0.69 Å RMSD to the product structures).[13]

Gutteridge and Thornton followed their work noted above by looking for conformational changes upon ligand binding in a larger set of structures. In their study of 60 enzymes, ~75% of holo-apo pairs had C_α_ RMSD of ≤ 1 Å. This RMSD was contrasted with the C_α_ RMSD observed among apo-apo protein pairs as a baseline, where ~83% of 31 apo-apo pairs had a C_α_ RMSD of ≤ 1 Å.[14]

Gunasekaran and Nussinov classified 98 proteins into three categories based on maximum C_α_ displacement between holo and apo structures: rigid proteins (≤ 0.5 Å), moderate (0.5 Å < and ≤ 2.0 Å), and flexible (> 2 Å).[15] All classes had the same contact density, so flexibility in certain residues was not due to loose packing. Rigid and moderately flexible proteins were seen to have more polar-polar interactions: 35% and 34% for rigid and moderately flexible versus 28% for flexible proteins. Overall, most of the φ and ψ changes between apo and holo were minimal. All classes had a few binding site residues with φ and ψ angles in poor regions of the Ramachandran map. There were more in apo than holo structures, and they tended to cluster near the binding site. Furthermore, they found no notable difference in SASA of the binding site residues of their three classifications of binding sites (rigid, moderately-flexible, and very-flexible).[15]

Brylinski and Skolnick found that most apo-holo protein pairs did not exhibit a significant structural difference and that holo-holo protein pairs exhibited even less change, using the C_α_ RMSD metric.[16] For 521 single-domain apo-holo structural pairs, 80% had an RMSD ≤ 1 Å, and among a set of single-domain holo-holo pairs, ~ 92% had an RMSD ≤ 1 Å.

Marks *et al*. found that the length of loop fragments in ensembles of sequence-identical protein structures was positively correlated with the likelihood of those loops demonstrating high levels of structural variation (localized backbone RMSD > 2Å).[17] However, the likelihood of demonstrating high structural variation was relatively low, even in longer loops (3.85% of their sampled 20-residue loops). This dataset involved 5548 unique protein sequences for which there were at least two X-ray crystal structures present in the PDB with resolution of 2.0 Å or better.

Qi and Hayward investigated 203 sets of enzymes with structures composed of domain pairs, both with and without functional ligands.[18] The ligands in these domain pairs were split into two groups based on whether they were within 4Å of both domains (150 ligands) or only one domain (53 ligands). They found that the dual-domain contacting ligands were often (84%) in contact with the “extended bending region” (the residues present between the annotated domains and three residues into each annotated domain). Conversely, the single-domain contacting ligands were rarely (13%) in contact with that extended bending region. This indicates that ligands which trigger domain motions *via* their binding event, and do not contact both domains (non-spanning trigger-ligands), rarely bind in close proximity to the bending region between the two protein domains. However, the authors of this study noted that the scope of their work is limited to large-scale “domain motions” as annotated by the source material they used.

Amemiya *et al*. established the Protein Structural Change DataBase (PSCDB)[19, 20], which focuses on larger-scale protein conformational changes, similar to the previously mentioned work by Qi and Hayward. PSCDB’s coverage extends beyond domain motions, into local subcomponents of domains, but not down to residue-level motions. Their presentation of protein motions is accomplished with atomic displacement and linear response theory of the domain sub-components using a dataset of 839 apo-holo protein pairs. Across their dataset, only 7% of their proteins displayed domain motion directly coupled to ligand binding, 15% displayed local (sub-domain) motion coupled directly to ligand binding, and 39% did not display any significant motions between the apo and holo state.

Fradera *et al*. found that the binding site’s structure is preserved upon ligand binding as evidenced by the fact that the average all-atom, binding site RMSD changes ≤ 1 Å, that more than 90% of atoms in contact with the ligand move less than 1 Å, and that most binding sites had only modest changes in their electrostatic potentials.[21] However, they found that these small movements were capable of inducing significant changes in volume and shape such that volume similarity indices (η) ranged from 0.44 to 0.90. The disparity in geometric similarity indices point to the need for other modes of analysis to accompany RMSD. These results hint that small changes in backbone displacement can result in greatly increased availability of side-chain conformational space.

### Side chain analysis

Analysis of side chains reveals additional qualities of protein flexibility and highlights the detriment of excluding side-chain motion in docking. In a validation study of the SLIDE docking tool, Zavodszky and Kuhn examined how many binding events could be modeled if an apo protein structure was only allowed minimal side-chain rotations.[22–24] They compared their flexible SLIDE docking tool to rigid docking with 20 different proteins (having 63 holo structures and 20 apo structures), where the backbone RMSD between the apo and holo structures ≤ 0.5 Å (thus no backbone changes would be necessary to dock the ligand). Only minimal side-chain changes were needed. SLIDE was able to dock all of the ligands within 2.5 Å RMSD of the crystal-structure pose while rigid docking only worked for 32 of the 63 structures. SLIDE changed 94% of the side chains by < 45° and 82% of the side chains less than 15°. This range of movement used in SLIDE is comparable to the natural variation observed among different holo crystal structures. Among the holo crystal structures in their set, 90% of the side chains changed by < 45°, and 75% changed by < 15°. Thus, small changes are typical, but more importantly, they are critical for accurate results in half of their studied protein structures.

Heringa and Argos have also described how ligand binding was sufficient to induce strain and push some binding-site side chains into rotamers outside of the typical minima.[4, 5] This supports the idea of rotameric changes being heavily influenced by ligand binding events.

Zhao, Goodsell, and Olson examined flexibility differences between amino acids.[25] They examined the variation of χ_1_ angles among different apo structures of the same protein to establish limits of natural variation in the side-chain χ_1_ of each amino acid. The authors established ranges for each amino acid that represent 90% of the observed conformations. Ile, Thr, Asn, Asp, and large aromatics showed limited flexibility, but Ser, Lys, Arg, Met, Gln and Glu were very flexible.

Najmanovich *et al*. examined side-chain flexibility upon ligand binding with their BPK database of 221 proteins containing 523 holo structures matched with 255 apo structures.[26] Overall, 94.4% of all χ_1_ angles changed less than 60°. In 40% of the apo-holo protein pairs, none of the χ_1_ values differed by more than 60°. However, the other 60% had at least one χ_1_ undergo a large conformation change beyond 60°. Rotations of 60° or greater in binding-site residue side chains are significant enough that most rigid docking will fail.[12, 24, 27] More importantly, many movements that are less than 60° will still be problematic. Therefore, less than 40% of these structures can be adequately treated without including flexibility. This study then showed that no correlation could be found between backbone movements (measured in the largest C_α_ displacement) and side-chain flexibility (measured as the fraction of side chains undergoing a change of ≥ 60°). This easily explains cases where C_α_ RMSD implies a protein is rigid, but χ-angle analysis reveals a flexible binding site.

Gaudreault, Chartier, and Najmanovich further explored side-chain flexibility utilizing their SEQ dataset, which contains 188 apo-holo protein pairs.[28] They concluded that at least one residue in the binding site undergoes significant rotameric change upon ligand binding in about 88% of their tested cases. At most, five rotamer changes account for all observed movements in 90% of their test cases, and rotamer changes are essential in 32% of flexible binding sites. The different amino acids were shown to have an 11-fold difference in their probability to undergo changes. There are two major takeaways from this work. First, at least one flexible residue is present in nearly all of the binding sites that they tested. Second, different amino acids have notably different propensities to undergo change in rotameric conformation.

### Current study

The previous studies reveal that there are often a few key residues with significant flexibility within binding sites of otherwise rigid residues that do not undergo significant rearrangement upon ligand binding. However, some of the studies noted above are limited to very small sets of proteins. Additionally, none of the studies covered all three comparison types: apo-apo, holo-holo, and apo-holo.[29] This is especially important because analysis of induced flexibility (apo-holo) has little relevance without first knowing the inherent variability in each structure type (apo-apo, holo-holo). While changes from ligand binding have been observed, they have not been appropriately separated from inherent variation in proteins.

This study aims to assess protein flexibility upon ligand binding, employing a large dataset and focusing on contrasting inherent flexibility to changes upon binding. Each protein in the dataset has at least two holo and two apo structures, so we may compare the observed variation observed in proteins with ligands (holo-holo pairs), without ligands (apo-apo pairs), and between the two sets (apo-holo pairs). We use a large and carefully created dataset so that the observed differences can be statistically quantified. This study describes a comprehensive set of 305 protein sequences, represented by 2369 holo and 1679 apo protein crystal structures. We describe statistically significant differences in flexibility upon ligand binding. To confirm these changes are truly due to flexibility, correlations to other properties such as ligand size and crystal-structure resolution were investigated.

## Results and discussion

### Dataset properties

The most recent release of Binding MOAD[30] was clustered to obtain relevant holo structures, matching apo structures were obtained from the RCSB Protein Data Bank (PDB)[31, 32] as described in the methods section. Upon filtering for proteins with at least 2 holo structures and 2 apo structures, this dataset reduces to 305 different proteins represented by 2369 holo structures and 1679 apo structures. An index of all apo and holo structures for each protein family in this dataset is provided as a CSV in the supporting information (S1 Table). These 305 proteins represent 284 different pfams[33], emphasizing the largely non-redundant nature of the set. Our dataset is over an order of magnitude larger than the previously utilized datasets for comparing apo to holo structures. Our dataset has relatively low redundancy with previously utilized datasets. For example, Skolnick’s dataset of 521 apo-holo protein pairs has only 69 apo and 49 holo structures in common with our dataset.[16]

The proteins with the most holo structures are carbonic anhydrase II followed by trypsin, with 174 and 120 holo structures, respectively. The proteins with the most apo structures are lysozyme followed by ribonuclease-A, which have 280 and 79 apo structures, respectively. This redundancy is accounted for by giving each protein family one overall value (a maximum, mean, or median) to describe all of its data in our analyses. However, we note that each structure in a family serves as a sampled state of conformational space. Therefore, families containing a larger number of structures will likely appear as more flexible simply due to the increased number of opportunities to sample different conformations.

The ligands in our dataset are diverse and represent many different classes of molecules. The average molecular weight of the ligands is 374 g/mol with 80% of ligands less than 500 g/mol and 95% less than 800 g/mol. The heaviest ligand is a seven-residue synthetic peptide bound to Endothiapepsin in structure 4LP9 at 999.18 g/mol. The smallest ligand is hydantoin which is bound to a zinc dihydropyrimidinase in crystal structure 4LCS and weighs 100 g/mol.

The apo proteins and holo proteins have average resolutions of 1.82 Å and 1.84 Å, respectively. Apo and holo median resolutions were 1.84 Å and 1.85 Å, respectively. There were 159 families better resolved in their holo form, 139 families better resolved in their apo form, and seven families with the same average resolution in both their holo and apo forms. Therefore, there is little bias between the resolution of the holo and apo sets that could influence the measurements used in this study.

### Unified binding sites

Traditional descriptions of ligand binding sites use residues within some distance cutoff of the ligand contained in a protein crystal structure. Our “unified binding sites” are a union of all residues within a 4.5 Å distance cutoff from *any* bound ligand within *all* the holo structures in a protein family (hydrogens were not considered). These unified binding sites represent the totality of the binding site. Unified binding sites averaged 21 ± 9 amino acids in size.

### Flexibility of protein backbones

**Fig 1:**
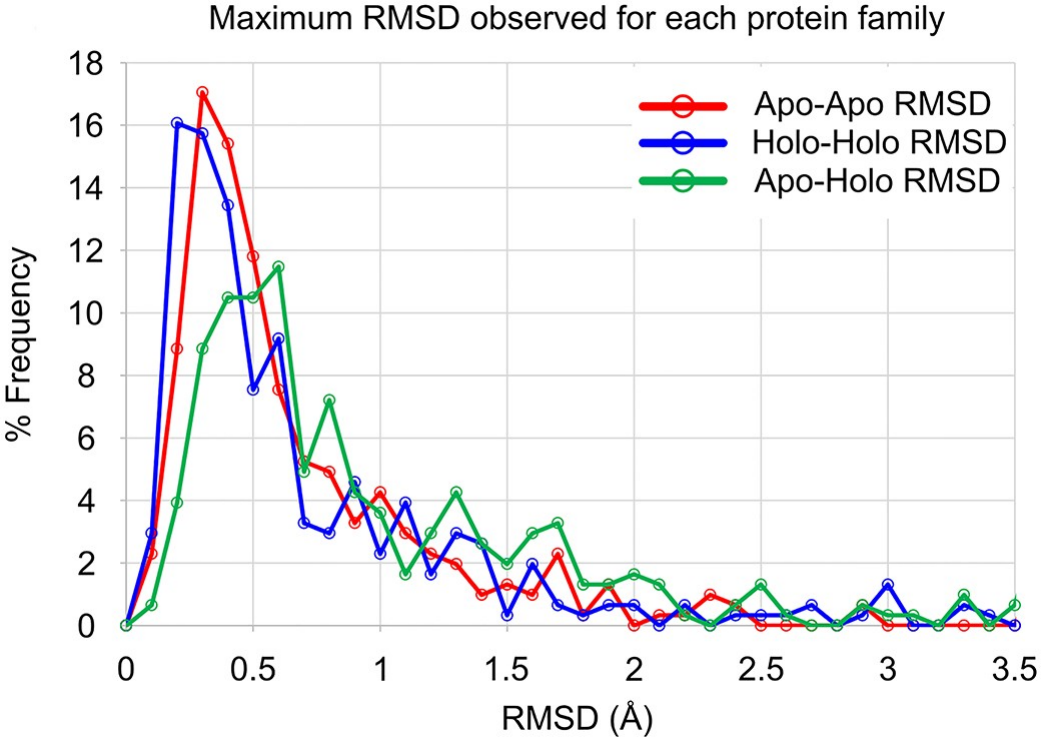
Distribution of maximum backbone RMSD for each protein family. The data for the apo-apo pairs is shown in red, holo-holo pairs are shown in blue, and apo-holo pairs are shown in green. There is no statistical significance to the difference in apo-apo vs holo-holo data (*p* > 0.05, difference in medians = 0.025 Å). The difference between the apo-holo data and apo-apo data are significant (*p* < 0.0001, difference in medians 0.241 Å), as is the difference between the apo-holo and holo-holo data (*p* < 0.0001, difference in medians 0.266 Å). It should be noted that only 5% of the families have apo-apo RMSD >2 Å, 6% of the families have holo-holo RMSD >2 Å, and 10% of the families have apo-holo RMSD >2 Å. This underscores the relatively low conformational flexibility seen in the backbones of at least 90% of the protein families.

**Fig 2:**
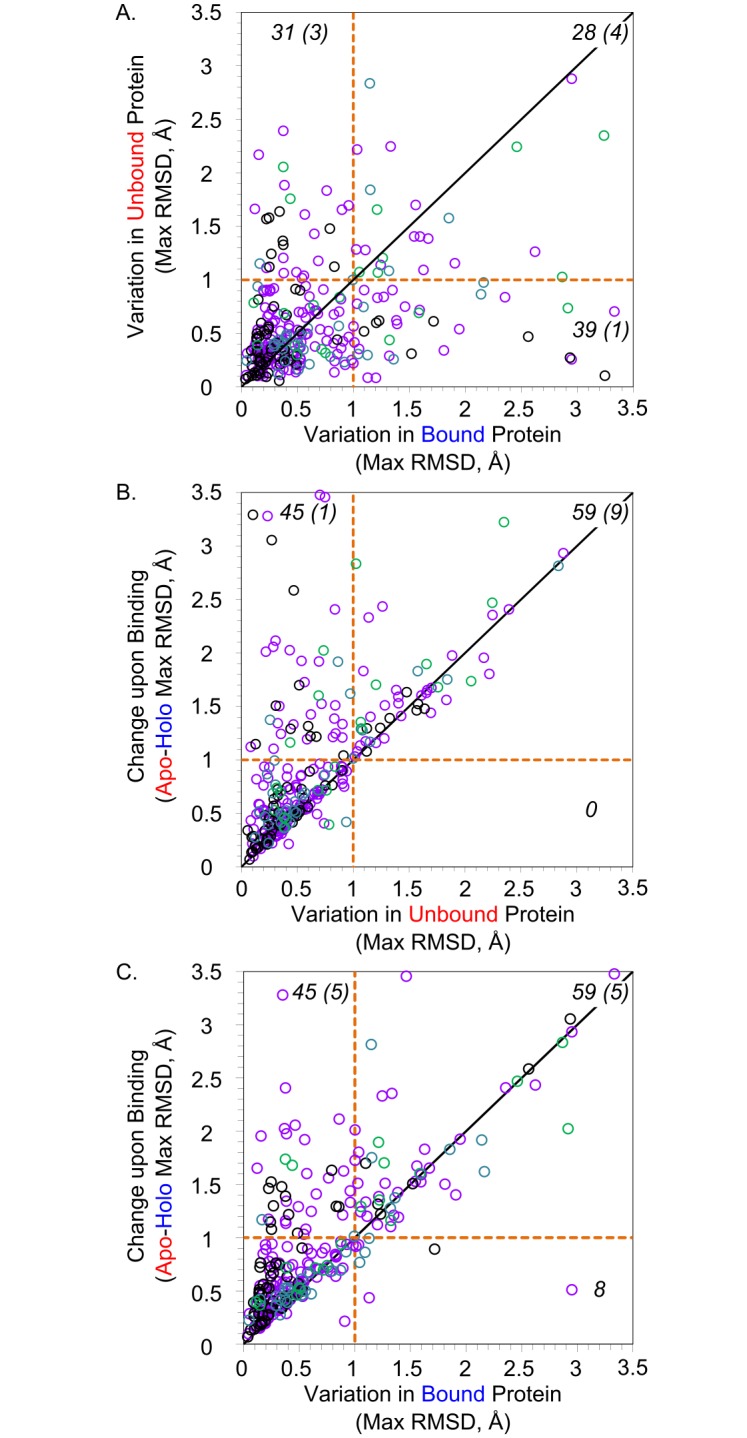
Analyses of maximum backbone RMSD for each protein family. Each point represents the maxima observed in one protein family, and the number of points of each section is labeled in black (numbers in parenthesis are points with values > 3.5 Å). A) The maximum across the apo-apo pairs is compared to the maximum of the holo-holo pairs; 207 proteins display RMSD ≤ 1 Å for both groups. B) The maximum across the apo-holo pairs is compared to the maximum of the apo-apo pairs; 201 proteins display RMSD ≤ 1 Å for both groups. C) The maximum across the apo-holo pairs is compared to the maximum of the holo-holo pairs; 201 proteins display RMSD ≤ 1 Å for both groups. Family data points are colored by the number of apo+holo structures in the family: black have 4 structures (67 families with 2 apo and 2 holo structures), purple have 5–14 structures (184 families), blue have 15–30 structures (32 families), and green have >30 structures (22 families).

Backbone RMSD overlays for the entire backbone of all structures of each protein were obtained and the maximum RMSD value for each type of pairing (e.g. apo-apo, apo-holo, holo-holo) within each family was determined (see Methods). Family maxima were chosen instead of medians or averages to readily identify proteins capable of large conformational changes. Table 1 presents the averages and medians of the maximum RMSD values for the 305 unique proteins. Distributions of the maximum RMSD are given in Fig 1. The maximum RMSD for the apo pairs, holo pairs, and apo-holo pairs are compared for each of the protein families in Fig 2.

**Table 1:** Averages and medians of the maximum backbone RMSDs.

	Average (Å)	Median (Å)
Apo-Apo Pairs	0.86	0.45
Holo-Holo Pairs	0.72	0.43
Apo-Holo Pairs	1.16	0.69

Apo structures and holo structures have similar conformational variation based on the comparison of the maximum apo RMSDs versus maximum holo RMSDs of each protein (Figs 1 and 2A). In general, proteins tend to have the same conformational flexibility within the apo and holo states. Only 10% of the proteins’ apo structures show significantly greater backbone flexibility than their holo structure counterparts, and 12% of the proteins’ holo structures show significantly greater backbone flexibility than their apo structure counterparts (31 apo families, 39 holo families). There were 28 families with both Apo and Holo maximum RMSD > 1 Å, indicating that both binding states are relatively flexible. The maximum backbone RMSD for apo and holo structures were both < 1 Å for 207 of the 305 proteins, showing that ~68% had negligible conformational flexibility regardless of ligand binding. Wilcoxon signed-rank tests support this, showing that apo vs. holo data distributions are not significantly different with *p* > 0.05 (see Methods).

As we expect, there is greater variation seen in going between apo-holo pairs (Figs 1 and 2B and 2C). Compared to apo-apo and holo-holo pairs, 15% of proteins (45 protein families) have significantly more conformational space available to their backbones between the unbound and the bound state (apo-holo pairs) when compared to either the apo (Fig 1B) or holo (Fig 1C) states. Importantly, these 45 protein families are not completely redundant between the two cases, sharing only 14 proteins in those 45.

Analyzing RMSD measurements across all proteins, the amount of conformational space available to apo proteins is not significantly different than that of holo proteins (*p* > 0.05) (Figs 1 and 2A, Table 1). Most notably, the amount of conformational space *between* apo and holo structures is greater than that *within* either the apo (*p <* 0.0001) or holo (*p <* 0.0001) protein sets (Figs 1 and 2B and 2C, Table 1). This suggests that the backbones in each of the apo and holo datasets occupy equally sized subsets of the total conformational space available, and there is a great deal of overlap between the two sets. While statistically significant, the difference of 0.86 Å RMSD in apo structures, 0.72 Å RMSD in holo structures, and 1.16 Å RMSD between all structures is less than 0.5 Å RMSD of change. This is likely negligible in the context of an entire protein structure and is close to experimental error, given B-factors for most backbone atoms.

A low global RMSD measurement can mask large displacements in very few residues. Therefore, RMSD values were also calculated specifically for the atoms within the unified binding sites to focus on localized changes incurred upon ligand binding. Binding-site backbone displacement is slightly greater than the whole backbone (Table 2). However, the distribution of RMSD by family and type remains largely unchanged (S1A, S1B and S1C Fig in Supporting Information). These results are observed for both the apo and holo structure subsets (S1D and S1E Fig) in Supporting Information.

**Table 2:** Averages and medians of the maximum backbone RMSDs for binding site residues only.

	Average (Å)	Median (Å)
Apo-Apo Pairs	1.19	0.31
Holo-Holo Pairs	1.16	0.36
Apo-Holo Pairs	1.80	0.59

Relationships between other metrics have also been investigated. Ligand size would logically impact the magnitude of protein-ligand contact area, and structure resolution can drastically affect our perception of a molecular environment, so it is appreciable to question whether or not these factors have impacted our results. R^2^ values between RMSD vs. ligand mass and RMSD vs. structure resolution were calculated to be < 0.02 at most, for all cases. This indicates that no corrrelation is observable between backbone motion and ligand mass or structure resolution.

### Conformational sampling of the side chains

Analysis of the protein backbone describes large-scale organizational changes in a protein structure, but it does not necessarily answer questions about atomic contacts with ligands. The most dynamic atoms of a protein are those of the side chains. These side-chain atoms have the highest propensity to adopt new conformations in order to interact with ligands. As such, investigating side-chain atoms is crucial. Traditional methods of characterizing side-chain behavior revolve around the dihedral angles of the side-chain atoms. The first dihedral angle (χ_1_) can be examined alone[25], or all available χ angles (depending on residue type) can be assembled to describe more detailed rotameric states.[24, 26, 28]

While the total rotameric states do provide more detail, they introduce two major issues. First, discerning between nitrogen and oxygen atoms in X-ray data is a known problem and a near impossibility without contextual data and intelligent processing. Many structures benefit from corrections and residue flipping to obtain more reasonable side-chain conformations even after they are deposited into the PDB. This is typically accomplished as a manual or semi-automated process, which is unfeasible with the size of dataset used in this work. Second, the more distal atoms of protein side chains have a tendency to have high B-factors and poorer electron density. This results in a lower inherent confidence in conjectures based on this data.

Due to these issues, we have elected to analyze our side chains purely by the χ_1_ angle (see Methods). The simplified approach of solely the χ_1_ angle may leave some flexibility undetected if the motions occur toward the terminal portions of the side chains. However, the χ_1_ angle has the largest impact on the occupied conformational space of any side chain. Thus, the motions observed from the χ_1_ angles indicate the most significant motions of side chains.

**Fig 3:**
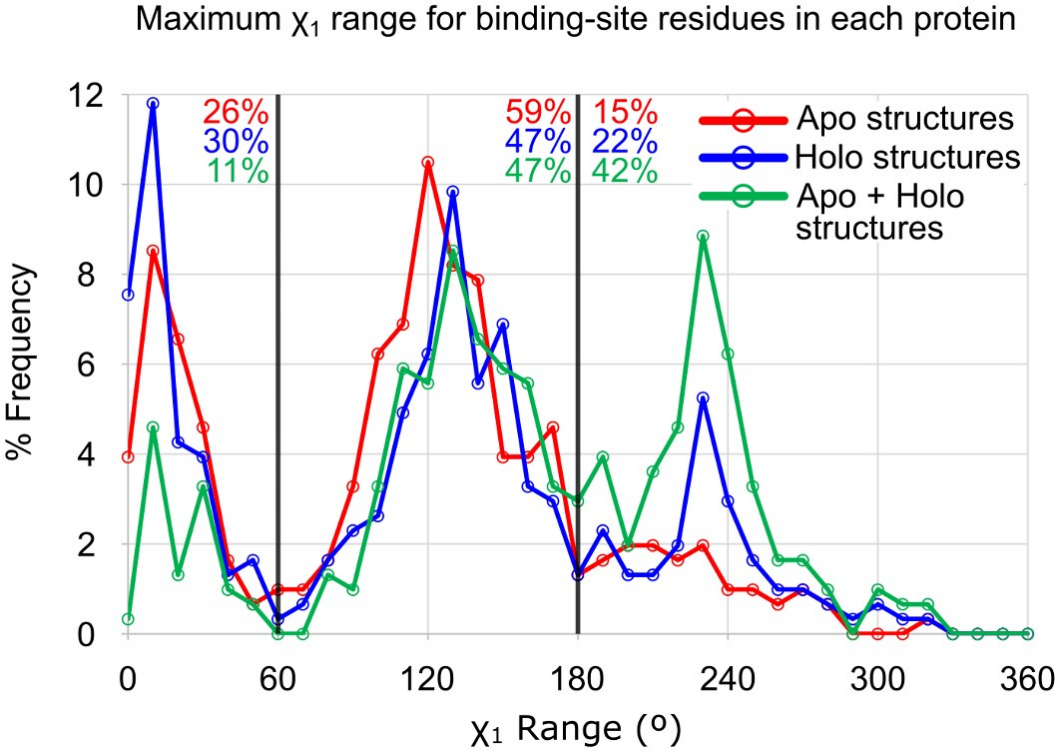
Distribution of the maximal χ_1_ range in each binding site. Again, the flexibility of the apo and holo states are approximately the same. When the structures are combined, much greater variation is seen in the maximum χ_1_ range. The ranges observed across the apo structures are shown in red, and the ranges across the holo structures are shown in blue. The line in green shows the χ_1_ ranges measured when the apo and holo structures are analyzed together (apo+holo). The population of structures with maximum χ_1_ ranges occupying one conformational well (0–60°), two wells (60°-180°), and all three wells (180°-360°) are given in red, blue, and green numbers for the apo, holo, and apo+holo analysis, respectively.

**Fig 4:**
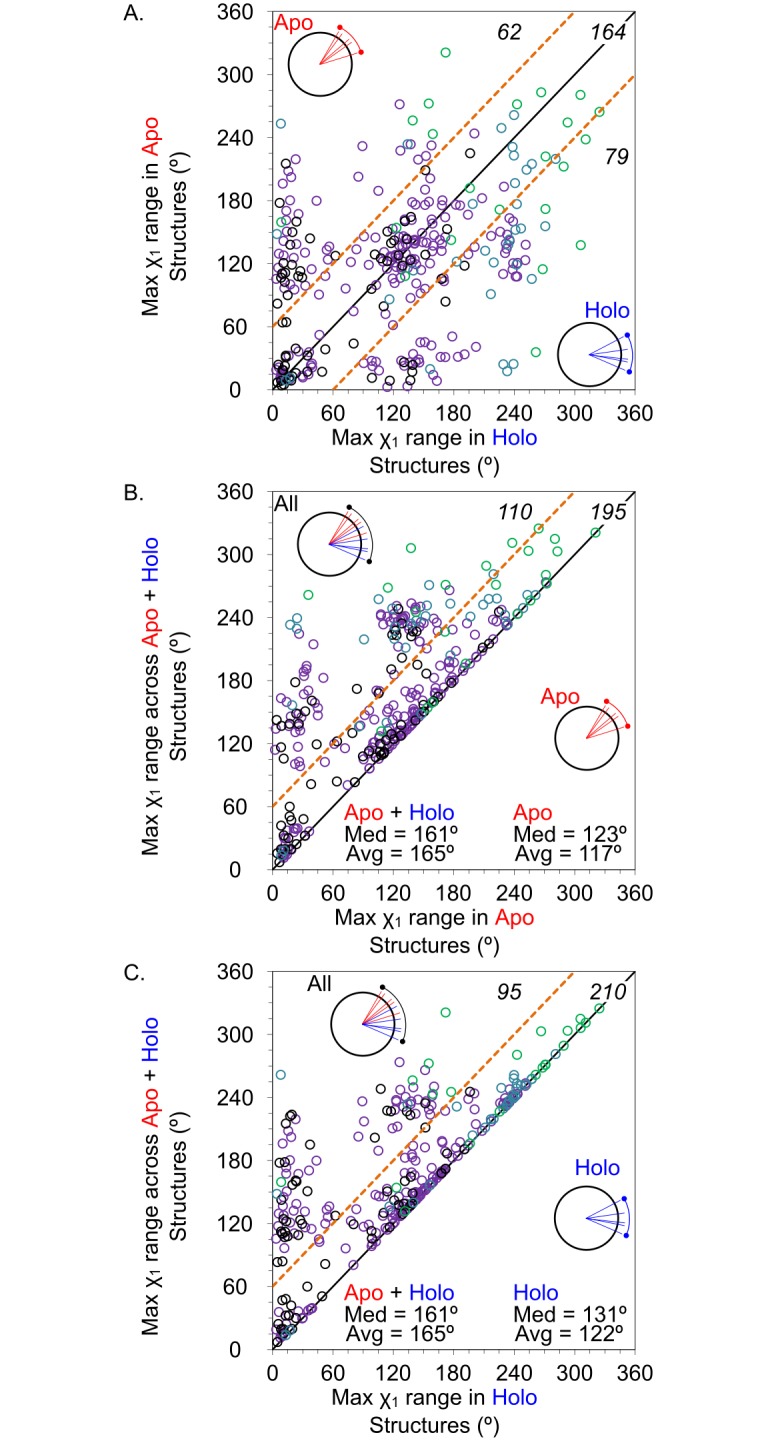
Comparisons of the maximal χ_1_ range in each binding site. For each protein family, the maximum χ_1_ range is given for A) apo vs holo structures, B) apo vs apo+holo structures, and C) holo vs apo+holo structures. The number of points of each section is labeled in black. Family data points are colored by the number of apo+holo structures in the family: black have 4 structures (67 families), purple have 5–14 structures (184 families), blue have 15–30 structures (32 families), and green have >30 structures (22 families). The small radar plots are simply visual aids to demonstrate the assembly of χ_1_ ranges for the different data groups.

We first calculated the χ_1_ angles for residues within the unified binding sites (see Methods). Comparing χ_1_ angles only describe the relative positions of the side chains, not necessarily a degree of flexibility. Therefore, we use the range of χ_1_ angles seen across all structures in a set as a metric for dataset-to-dataset comparisons (see Methods). Comparing χ_1_ angle ranges yields information about the extent of occupied conformational space across sets of structures, like all apo structures, all holo structures, or apo and holo structures combined (apo+holo). Distributions of the maximum χ_1_ angle ranges are given in Fig 3. The maximum χ_1_ angle ranges for the apo structures, holo structures, and apo+holo structures are compared for each of the 305 protein families in Fig 4. It should be noted that some of the increase in χ_1_ ranges may come from the apo+holo set simply having more structures than the apo or holo set alone.

The distribution of maximum χ_1_ angle ranges shows the most variable side chain for each protein’s binding site. The trimodal distribution comes from those side chains occupying one, two, or three of the conformational wells around the χ_1_ angle. It is clear that the majority of apo and holo sets have at least one χ_1_ angle that spans two conformational wells (ie, the population from 60–180° is largest for apo and holo sets). Only 26% of apo structures, and 30% of holo structures have χ_1_ ranges that represent only one energy well (≤ 60°). When the two sets are combined (the green line for apo+holo in Fig 3), there is a significant increase in the number of proteins where the most flexible residue has a χ_1_ angle range that spans all three conformational wells available (ie, the population >180°). This shows that in going from the holo to apo state, many systems have side chains pushed into new conformational states not observed in the holo state. This is perhaps better seen in Fig 4B and 4C where roughly one third of the systems show significant displacement of their χ_1_ angles (apo+holo χ_1_ angle ranges increase by ≥60°).

**Fig 5:**
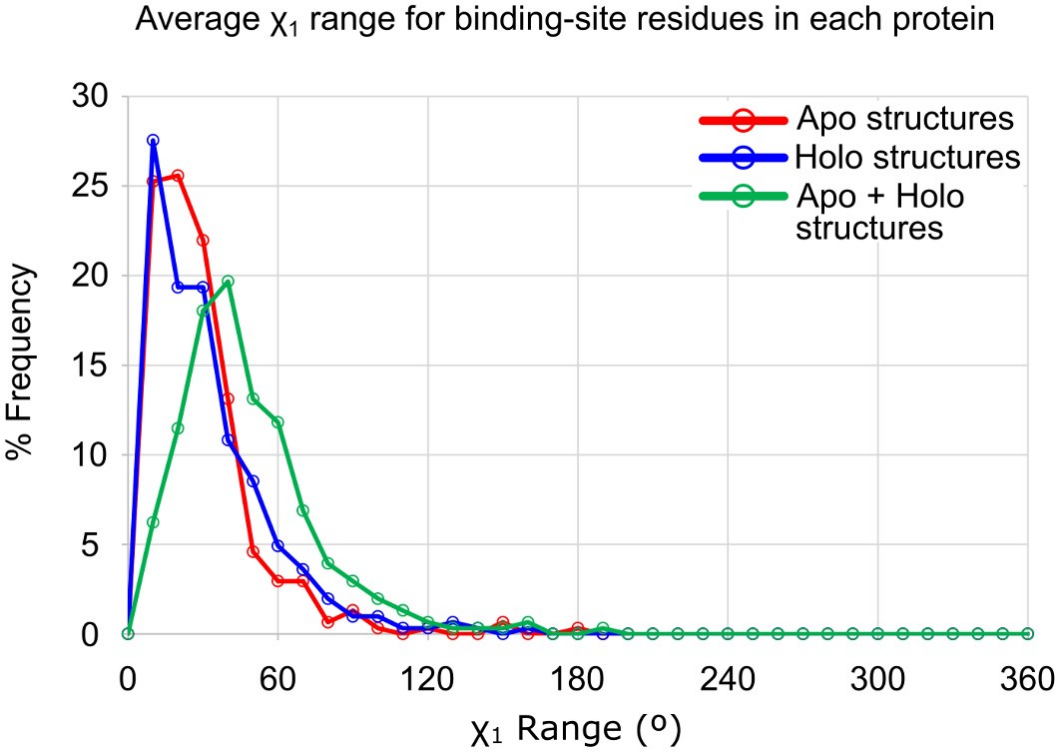
Distribution of the average χ_1_ range in each binding site. The ranges observed across the apo structures are shown in red, and the ranges across the holo structures are shown in blue. The line in green shows the χ_1_ ranges measured when the apo and holo structures are analyzed together (apo+holo). The medians of the average χ_1_ range are 19° for the apo structures, 21° for the holo structures, and 37° for the apo+holo structures. The flexibility of the apo and holo states is approximately the same with no statistical significance in their difference (*p* > 0.05). When the structures are combined, much greater variation is seen in the maximum χ_1_ range. The difference between the medians of the apo+holo and apo structures is 18° (*p* < 0.0001), and the difference to the holo structures is 16° (*p* < 0.0001).

Traditional statistical tests are not appropriate for the data on maximal χ_1_ ranges because the distribution is trimodal. If we examine the average χ_1_ ranges for each protein, the data are near-normal in their distribution and appropriate for Wilcoxon signed-rank tests. The median binding-site residue in holo structures exhibits a χ_1_ range of 21°, while the χ_1_ range median 19° in apo structures. The ranges of side-chain motion in holo structures and apo structures are statistically indifferent (*p* > 0.05, Fig 5). This follows the trend seen in the RMSD calculations, where the amount of available conformational space to apo structures is approximately the same size as the amount for holo structures. More importantly, the median χ_1_ range when combining all structures (holo+apo) is 37° (*p* < 0.0001 compared to both apo and holo datasets). This larger range of χ_1_ values for all (apo + holo) structures, as opposed to the corresponding apo or holo sets alone, suggests that ligand binding induces rotameric changes in side-chain orientations beyond the threshold of inherent variation.

Again, relationships to ligand size and structure resolution were investigated. All R^2^ values for χ_1_ range vs. ligand size and χ_1_ range vs. structure resolution were < 0.03, indicating that no correlation exists between these factors.

### Correlation between backbones and side chains

Correlations between backbone and side-chain motion were assessed by calculation of R^2^ values between appropriate datasets using JMP.[34] Comparison of the maximum RMSD vs. maximal χ_1_ range for apo-apo pairs, holo-holo pairs, and apo-holo pairs yielded poor R^2^ values of 0.02, 0.16, and 0.04, respectively. Lack of correlation between backbone RMSD and χ_1_ range suggests that the flexibility of protein backbones and side chains are independent.

### Flexibility of individual amino acids in the binding sites

Establishing that significant changes in side-chain orientation occur upon ligand binding inspired an investigation of the χ_1_ angles on a per-amino-acid basis. Radar plots of the occupied χ_1_ angles for each amino acid type across the binding sites of all proteins utilized in this study were generated (See S2A–S2R Fig in Supporting Information). The χ_1_ angles are distributed into three energy wells, with the largest population present where the side chain is gauche only to the N-terminal direction of the backbone. This case is exceedingly prevalent for any amino acids capable of forming a intramolecular hydrogen bond between its side chain and backbone nitrogen. The least common orientation places the side chain gauche to both the N- and C-terminal directions of the backbone, which is a very high energy conformation. Overall, this data shows that side chains in ligand-bound binding sites do not occupy exclusively different conformational space than unbound structures. The larger χ_1_ range resulting from calculating with all apo and all holo structures combined simply implies that there are rotameric changes occurring upon ligand binding.

**Fig 6:**
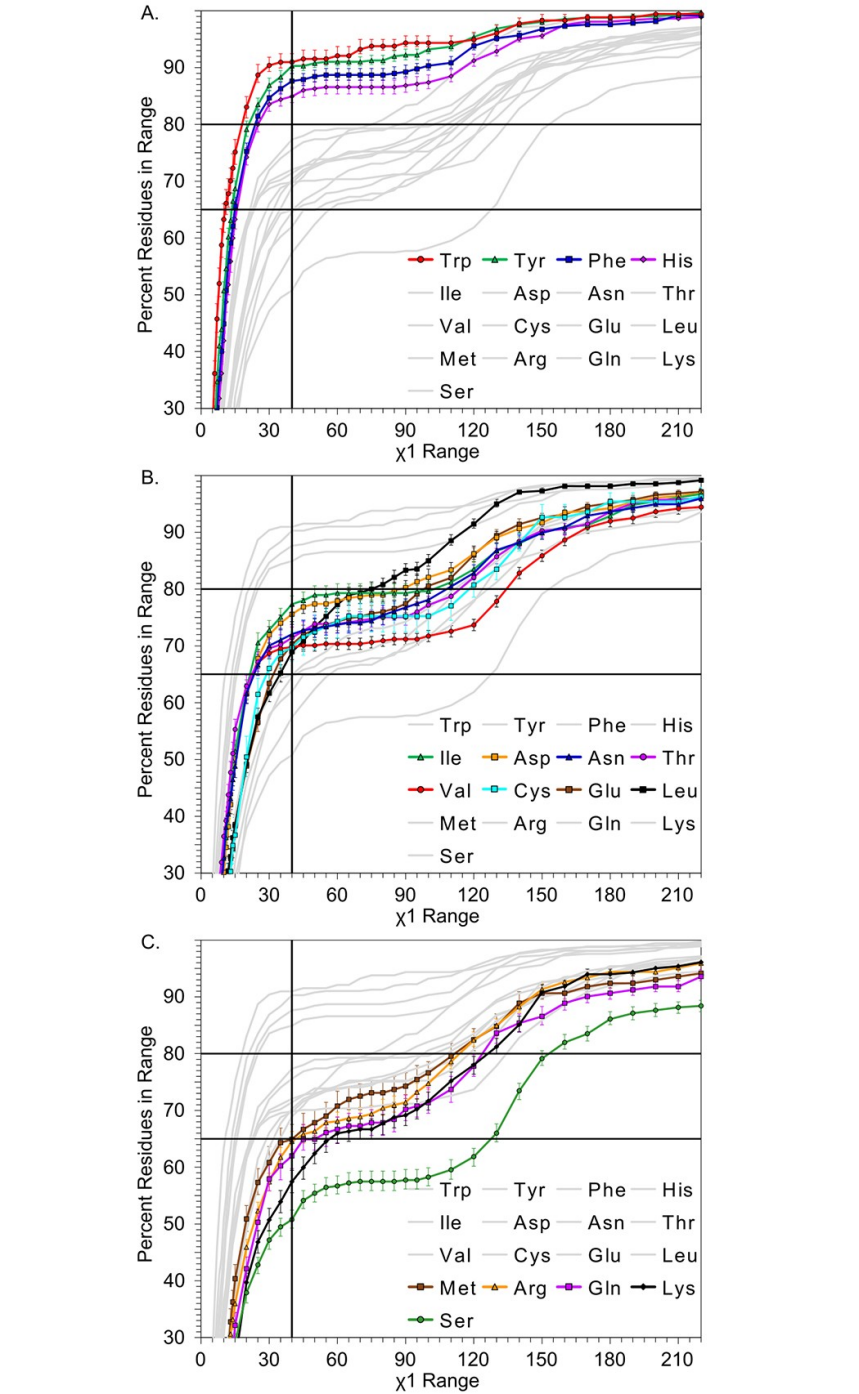
Cumulative distributions of binding-site χ_1_ ranges for each type of amino acid. The data describes the flexibility of different amino acids as a gradient of rotameric state change. Separated into three groups: A) rigid residues, B) semi-flexible residues, and C) very flexible residues. Error bars represent 95% confidence intervals.

The cumulative distributions in Fig 6 display the inherent flexibility of each amino acid type within binding sites of all structures. Important guidelines for incorporating protein flexibility in structure-based drug design may be extracted from the trends in these figures. If residues were allowed to sample 30° of χ_1_ conformational space, between 47–90% of side-chain variation could be captured, depending upon the residue type (most flexible Ser and most rigid Trp). In another perspective, trying to capture 90% of all variation would require only about 40° of sampling for the most rigid residue(s), but the most flexible would have to be allowed over 200° of sampling. To represent 90% of the variation in Ser would require 240° of motion, which is close to the complete range of motion between the three energy wells.

Using this type of breakdown, we rank the amino acids (from least flexible to most flexible) Trp, Tyr, Phe, His < Ile, Asp, Asn, Thr, Glu, Val, Cys, Leu < Met, Arg, Gln, Lys, Ser. Other studies have shown very similar trends with serine and lysine being flexible and the large, bulky amino acids such as tryptophan being rigid, although the ranking is not exact.[25, 26, 35] We determine this trend by observing the relative amounts of χ_1_ angle range representation at 40° and separating the data into rigid/semi-flexible/very flexible based on where there is overlap in the 95% confidence intervals. This trend coincides with classical biochemical intuition, where large hydrophobic residues are more sterically constrained. Not all large polar or charged residues show the same degree of flexibility in χ_1_.

This information is immediately applicable in flexible protein docking. Using different thresholds of data inclusion (i.e. what χ_1_ range is accommodated with 60% of some residue’s data), restrictions could be placed on residues during flexible docking relative to their starting positions (rotameric flip allowed or not). The occupation of observed χ_1_ angles can be used to find “forbidden” rotameric states. Leucine, for instance, almost never occupies the energy well characterized by two gauche interactions with the backbone (2.92% of apo data, 3.41% of holo data in S2J Fig in Supporting Information).

### Solvent accessible surface area

**Fig 7:**
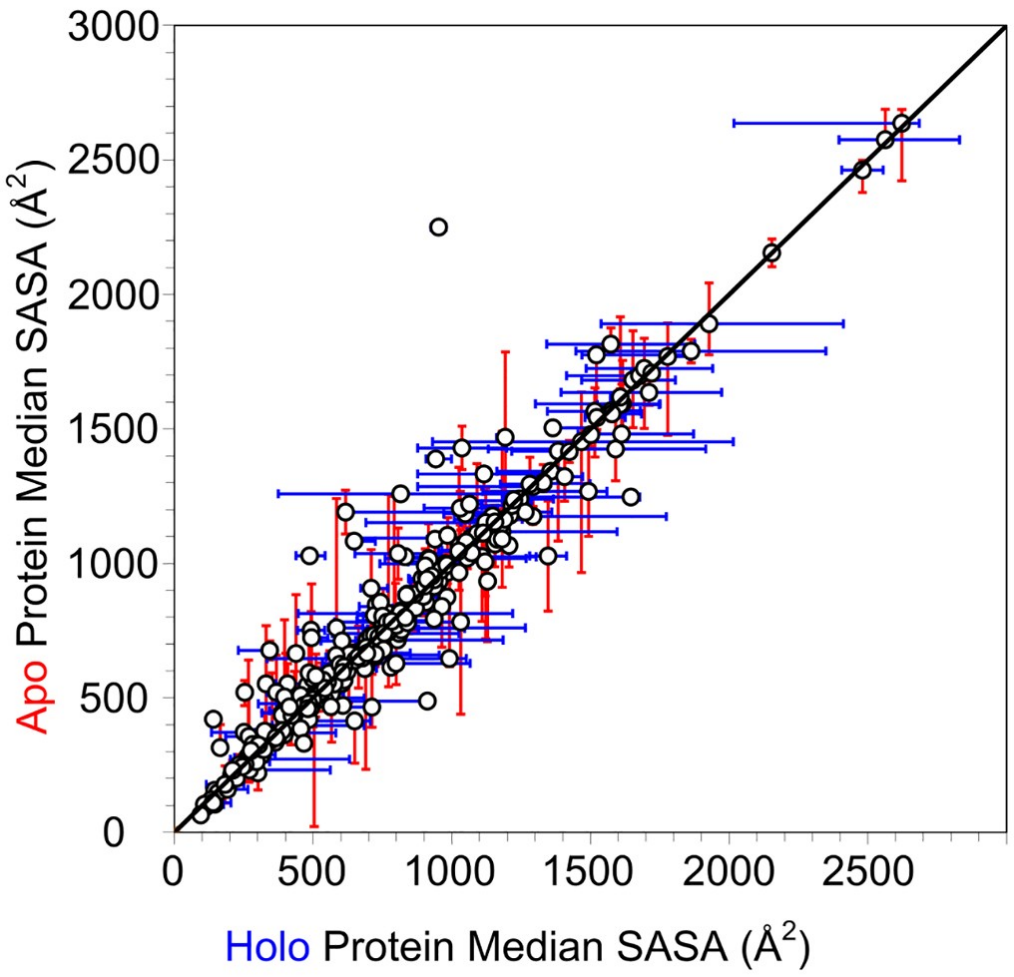
Median solvent accessible surface area of unified binding-site residues: Apo structures vs. holo structures. Error bars represent the minimum and maximum SASA value in each family for each structure type. The outlier is adenylate kinase, which exhibits significant domain motions upon ligand binding.

**Fig 8:**
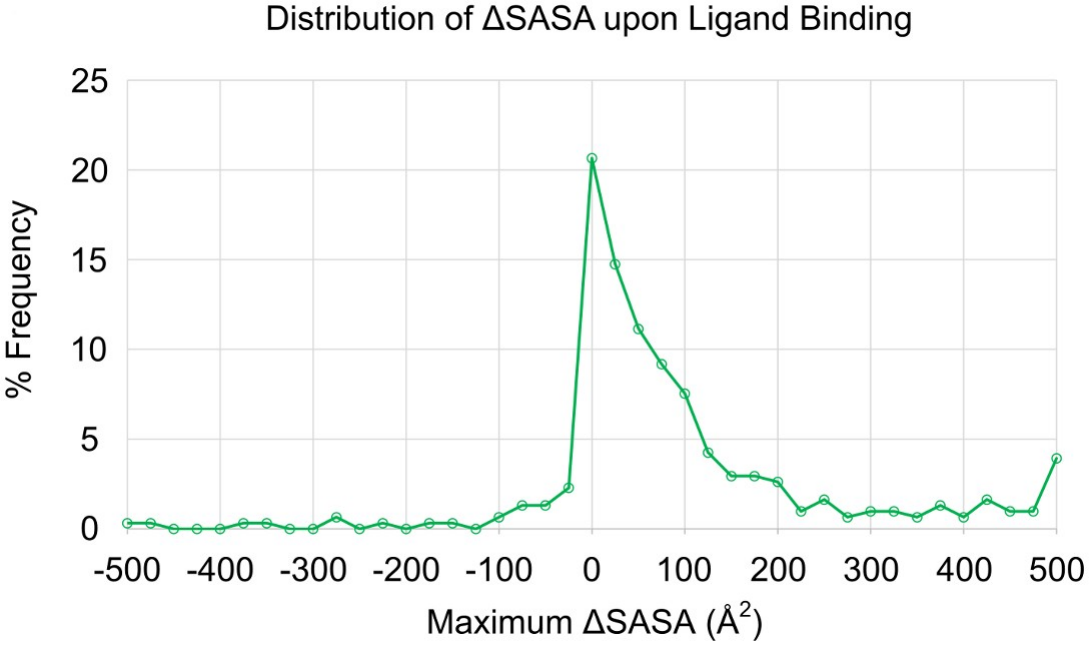
Distribution of the maximum change in solvent accessible surface area of unified binding-site residues. ΔSASA was calculated as maximum Holo SASA—minimum Apo SASA.

SASA calculations have been applied to describe protein-protein binding events[36, 37], as well as physicochemical properties of biologically relevant ligands.[38] Fig 7 displays the median, minimum, and maximum SASA values of the unified binding sites for apo structures and holo structures within each of the 305 protein families. There does not appear to be any significant difference observed in SASA for holo structures against their apo counterparts. Only one protein in our set (adenylate kinase) seems to have significantly more SASA in its apo state, which is caused by a large partial domain movement when no ligands are bound. A distribution of ΔSASA between the minimum SASA apo structure and maximum SASA holo structure for each family is presented in Fig 8. The great majority of proteins (72%) have ΔSASA ≤ 100 Å^2^, which is rather small. Only 9% of all proteins lose SASA upon binding ligands (ΔSASA < 0 Å^2^). These findings agree with Gunasekaran and Nussinov’s results suggesting no distinguishable changes in SASA upon ligand binding for flexible, semi-flexible, or rigid proteins.[15]

### Conclusions

Understanding protein flexibility is important in drug design, especially as crystal structures become more widely used as models for binding prediction.[39] This study examines how ligand binding influences protein flexibility. More specifically, it uses a large collection of proteins that have at least two holo and two apo structures to examine backbone and binding site variation among holo or apo structures inherently, as well as what differences arise from ligand binding.

We have shown that ligand-free structures and ligand-bound structures have nearly identical amounts of structural variation, in terms of residual backbone motion (measured in RMSD). A similar range of motion was seen in both the global and binding-site backbones for both the apo and holo structure subsets. The apo-holo pairs showed only slightly larger RMSD.

Examining the side chains through χ_1_ angle ranges reveals that apo structures and holo structures have roughly the same flexibility. However, when apo and holo states are combined, the χ_1_ angle ranges significantly increase, displaying that binding sites frequently have at least one side chain that gets pushed into new conformations in the presence of ligands.

Through the significant variance in observed side-chain conformations, and relative lack of backbone motion, we support a model of ligand binding where backbone motion is minute and side-chain flexibility is essential. The lack of correlation between the backbone and side-chain data further suggests that sampling large amounts of conformational space with protein side-chains is not necessarily coupled to having a flexible backbone. Combining these ideas indicates that addressing side-chain flexibility separately from backbone motion is appropriate, which agrees with many modern approaches to flexible ligand docking. Furthermore, it may also apply to the use of homology models in docking, where there is greater uncertainty in side-chain positions than in backbone positions. Allowing side-chain flexibility in these cases is likely essential.

## Methods

### Ethics statement

No humans or animals were used in this research.

### Holo dataset

The non-redundant holo structure dataset was derived from Binding MOAD, a source of high quality, protein-ligand X-ray crystal structures resolved at 2.5 Å or better.[30] Biologically relevant ligands are differentiated from opportunistic binders (e.g. salts, buffers, phosphate ions) in the crystal structures of Binding MOAD, making curation of relevant ligand structures straightforward. Furthermore, use of Binding MOAD excludes covalently attached ligands. Structures with more than one valid ligand were excluded from this study in favor of binary protein-ligand complexes to ensure that only one pocket was being analyzed in each protein. Any structures containing additional molecules in their binding site, such as additives, were also excluded.

Each structure in the holo set was clustered based on the sequence identity using stringent criteria of 100% sequence identity in both directions. A subsequent 95% sequence identity clustering of those families was then performed to suggest any families that should be merged due to simple N or C terminal amino acid additions. Sequence identity between structures was determined using BLAST.[40] Any families differing in protein core sequence were kept separate. Families containing fewer than two holo structures were removed. An index of all apo and holo structures for each protein family in this dataset is provided as a CSV in the supporting information (S1 Table).

### Apo dataset

A set of apo structures was compiled using the following protocol. The PDB[32] was screened for structures of 2.5 Å resolution or better which shared 100% sequence identity with one of the proteins contained in the holo dataset. The HET contents of these harvested structures were then carefully assessed to ensure they were appropriately “apo” or “ligand-free” structures with regards to their binding sites. Waters (HOH HETs) were considered valid components of Apo structures. HETs contained in the structure files were required to have a molecular weight ≤ 100 Daltons or be present in appropriate filtering lists derived from the curation of Binding MOAD[41]. The curation lists employed include the sugars, small organic molecules, membrane components, small metabolites, salts, buffers, solvents, crystal additives, cryoprotectants, detergents, and metal ions lists. A summary of these lists (620 HET groups) can be found in the supporting information (S2 Table). These various HETs were permissible in the structure files, so long as they were not present in the binding site. Any structures containing HET material apart from water (HOH) within 4.5 Å of any unified binding site residue were removed. After binning these apo structures into their appropriately matched 100% sequence identical protein family, families containing fewer than two apo structures were removed. An index of all apo and holo structures for each protein family in this dataset is provided as a CSV in the supporting information (S1 Table).

### File setup and preparation

These steps were taken prior to any calculations. The first biounit model containing the relevant ligand of the corresponding PDB structure was used by default for each structure. All hydrogens were removed from the files. Ligand data was extracted, and then all ligands were removed from the files. Waters were also removed.

All protein systems were renumbered utilizing the pdbSWS prior to binding site calculation and assembly.[42] If the entire length of a protein chain was not completely renumbered (e.g. the pdbSWS mapping starts at residue #70), the resulting binding-site contacts for the holo structures were examined to determine if this were to impact the resulting unified binding sites. There were no cases in which the partial renumbering was an issue.

In the cases where the pdbSWS templates would result in more than one numbering pattern inside of a family, one structure’s pdbSWS numbering was applied to the other structures. This was only possible after sequence alignment with EMBOSS: Needle[43] (standard parameters) to determine appropriately matching chains, and only if those chains had the same starting numbering to begin with. If there was no method to renumber a structure to the same pattern as the rest of its family aside from manually renumbering it, it was discarded from the dataset out of consideration for reproducibility.

Renumbering structures was necessary because some structures were numbered differently (especially common when going between apo and holo structures). Protein numbering becomes critically important in the case of unified binding sites, where it is necessary to harvest residue data from the site when there are no ligands present to define the site (apo structures).

### Ligand size

The molecular weight for each ligand was extracted from Binding MOAD. A unique feature of this set is the size of the ligands involved. This dataset allows for ligands composed of more than one HETATM group from the crystal structure. This study allowed peptides up to 10 amino acids, nucleotides up to 4 nucleic acids, and other multi-HET ligands. Multi-HET ligands were appropriately treated as one large molecule. For example, the inhibitor Aeruginosin98-B, in the PDB structure 1AQ7 of bovine trypsin, is comprised of the HET groups “34H+DIL+XPR+AG2”. Newer HET groups have been made recently that combine some of these multipart ligands, but these were not yet implemented to the PDB at the time of this analysis.

### Binding site identification and compilation of the “unified” binding sites

Each site was defined to include all protein residues within 4.5 Å of the biologically relevant ligands, which should capture both hydrogen-bonding and van der Waals interactions. Hydrogen atoms were not considered in the distance calculation for either the protein or the ligand. Most of the crystal structures for a given protein had different ligands bound, so many could have a slightly different set of residues near the ligand. Therefore, the summation of all sets of residues in all complexes for each protein was used to identify the union set if the binding pocket for that protein or its unified binding site.

### Maximum RMSD and χ-angle range calculations

In order to compare the overall similarity of all the structures of a protein, we calculated a maximum RMSD. RMSDs are pair-wise comparisons, and our analysis compared all structures of the same protein to one another. The RMSD calculations were based on all C_α_ in the backbone of the protein. Methods established previously in our lab were used to compute standard RMSD.[44] Binding-site RMSD values were also calculated for the unified binding sites.

To examine the flexibility of the side chains, χ_1_ was measured for residues (except Gly, Pro, and Ala) utilizing an in-house Perl script. Valines are represented by both of the two available χ_1_ angles, as Valine is symmetrical at atom γ for the calculation. Isoleucine also contains two χ_1_ angles, but the angle used in this analysis is that of the longer carbon chain.

Binning for χ_1_ angle plots was accomplished *via* an in-house Perl script. Data for each amino acid was binned on a per-residue, per-family basis, and then averaged over the total number of that residue in the entire dataset. For example, there are 405 Arg residues in the 305 binding sites, each of those are binned with their corresponding data, and then the bins for each of the 405 residues are averaged together to represent all Arg residues in the dataset.

The variation for a given residue was measured by determining the *range* of χ_1_ values observed for each residue in each binding site. This range is the smallest mathematical angle that contains all χ_1_ angles observed for each amino acid. (e.g. values of 30°, 45°, and 100° would yield a χ_1_ angle range of 100–30 = 70°.)

### SASA calculations

SASA was calculated using NACCESS.[45] Default probe size was used. All hydrogens, ligands, water, and HET groups were removed prior to calculation. This is default behavior for HET groups; however in the case of peptide ligands, it is necessary to remove the ligand from the file. SASA was calculated for all residues of the protein sequences first, and then the binding sites were extracted for analysis.

### Crystallographic data issues

Working with crystallographic data presents a number of problems for calculation-based studies. Below, we detail our process for handling these various problems.

For residues and ligands with multiple occupancies, only one orientation was used. The orientation chosen corresponded to that with a higher % occupancy or, in the case of a tie, the first listed orientation.

Addressing residues with missing coordinates proceeded as follows: For RMSD calculations, residues with missing coordinates were ignored. For χ_1_ calculations, residues with missing individual atoms were kept as long as calculation of the χ_1_ angle was not hindered by the missing atoms. In the case that critical atoms for χ_1_ calculation were missing or an entire residue was missing, the residue would be removed from the χ_1_ analysis (for all structures in that family). The unified binding sites across the 305 protein families included 6538 unique sequence residues, after removing 181 total residues (2.7% of the initial unified-binding-site residues) for resolution-related issues. In conclusion, these issues were relatively rare.

### Statistical methods

To assess if an observed difference between two groups (being apo, holo, or the entire apo-holo dataset) is sufficient to reject the null hypothesis that the two groups have identical distributions, Wilcoxon signed-rank tests were used (performed in JMP).[34] These tests applied to the distribution of maximum RMSD measurements among the families and average χ_1_ angles.

In lieu of that manner of statistical test, error bars describing 95% confidence intervals for Fig 6 were jack knifed by 1000 resamples of the data at 90% of the original dataset’s size or “leaving 10% out.”

All statistical analyses were performed utilizing the statistical packages JMP and R.[34, 46]

## Supporting information

S1 Fig Analyses of maximum backbone RMSD for only unified binding site residues within each protein family. Each point represents the maxima observed in one protein family, and the number of points of each section is labeled in black (numbers in parenthesis are points with values > 3.5 Å). A) The maximum across the apo-apo pairs is compared to the maximum of the holo-holo pairs, binding site residues only; 207 proteins display RMSD ≤ 1 Å for both groups. B) The maximum across the apo-holo pairs is compared to the maximum of the apo-apo pairs, binding site residues only; 201 proteins display RMSD ≤ 1 Å for both groups. C) The maximum across the apo-holo pairs is compared to the maximum of the holo-holo pairs, binding site residues only; 201 proteins display RMSD ≤ 1 Å for both groups. D) The maximum across the apo-apo pairs for only binding-site residues is compared to the whole backbone maximum for apo-apo pairs; 227 proteins display RMSD ≤ 1 Å for both groups. E) The maximum across the holo-holo pairs for only binding-site residues is compared to the whole backbone maximum for holo-holo pairs; 214 proteins display RMSD ≤ 1 Å for both groups.
(DOCX)

S2 Fig Radar plots of χ_1_ angle distributions. Distribution of χ_1_ angles observed in unified binding site residues. Values were normalized on a per-family basis before radar binning such that each unique protein sequence is represented equally, regardless of family size. Data for: A) All unified binding-site residues, B) Arg, C) Asn, D) Asp, E) Cys, F) Gln, G) Glu, H) His, I) Ile, J) Leu, K) Lys, L) Met, M) Phe, N) Ser, O) Thr, P) Trp, Q) Tyr, R) Val.
(DOCX)

S1 Table Comma separated file of every protein structure used in this analysis. The structures are broken down by protein family number and apo/holo distinction.
(CSV)

S2 Table List of permissible HET groups in Apo structures outside of the unified binding sites. (CSV)

S1 Text A zipped file of all the analysis scripts used to measure the RMSDs and χ_1_ angles. (ZIP)
