# Supplementary material for: Inherent versus induced protein flexibility: Comparisons within and between apo and holo structures
Source: PLoS Comput Biol. 2019 Jan 30;15(1):e1006705. doi: 10.1371/journal.pcbi.1006705 (PMC6370239; doi:10.1371/journal.pcbi.1006705)
Supplement: S1 Fig — Each point represents the maxima observed in one protein family, and the number of points of each section is labeled in black (numbers in parenthesis are points with values > 3.5 Å). A) The maximum across the apo-apo pairs is compared to the maximum of the holo-holo pairs, binding site residues only; 207 proteins display RMSD ≤ 1 Å for both groups. B) The maximum across the apo-holo pairs is compared to the maximum of the apo-apo pairs, binding site residues only; 201 proteins display RMSD ≤ 1 Å for both groups. C) The maximum across the apo-holo pairs is compared to the maximum of the holo-holo pairs, binding site residues only; 201 proteins display RMSD ≤ 1 Å for both groups. D) The maximum across the apo-apo pairs for only binding-site residues is compared to the whole backbone maximum for apo-apo pairs; 227 proteins display RMSD ≤ 1 Å for both groups. E) The maximum across the holo-holo pairs for only binding-site residues is compared to the whole backbone maximum for holo-holo pairs; 214 proteins display RMSD ≤ 1 Å for both groups. (DOCX) [file pcbi.1006705.s001.docx]

**
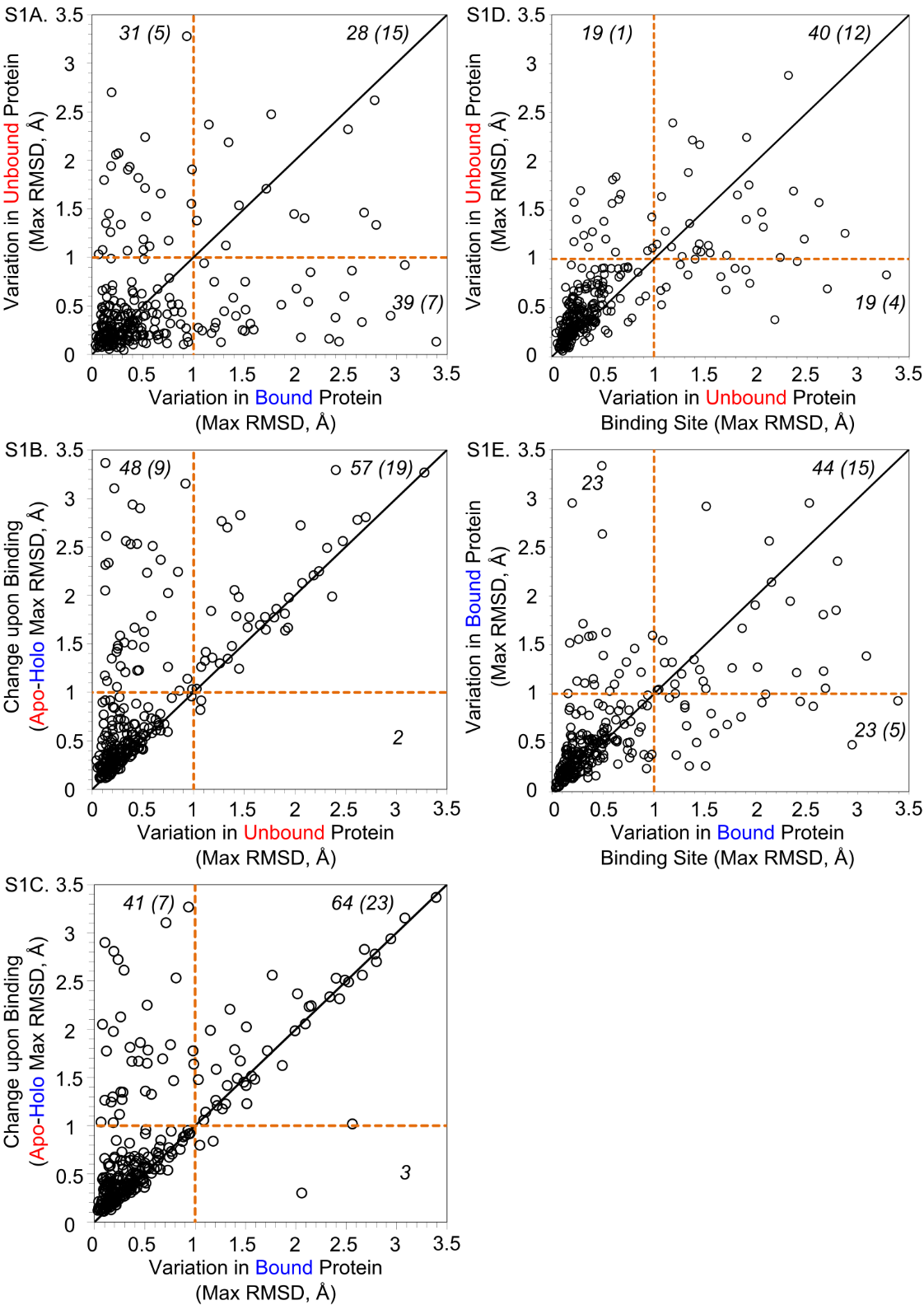
**

**Figure_S1.** **Analyses of maximum backbone RMSD for only unified binding site residues within each protein family.** Each point represents the maxima observed in one protein family, and the number of points of each section is labeled in black (numbers in parenthesis are points with values > 3.5 Å). A) The maximum across the apo-apo pairs is compared to the maximum of the holo-holo pairs, binding site residues only; 207 proteins display RMSD ≤ 1 Å for both groups. B) The maximum across the apo-holo pairs is compared to the maximum of the apo-apo pairs, binding site residues only; 201 proteins display RMSD ≤ 1 Å for both groups. C) The maximum across the apo-holo pairs is compared to the maximum of the holo-holo pairs, binding site residues only; 201 proteins display RMSD ≤ 1 Å for both groups. D) The maximum across the apo-apo pairs for only binding-site residues is compared to the whole backbone maximum for apo-apo pairs; 227 proteins display RMSD ≤ 1 Å for both groups. E) The maximum across the holo-holo pairs for only binding-site residues is compared to the whole backbone maximum for holo-holo pairs; 214 proteins display RMSD ≤ 1 Å for both groups.
