# Supplementary material for: Inherent versus induced protein flexibility: Comparisons within and between apo and holo structures
Source: PLoS Comput Biol. 2019 Jan 30;15(1):e1006705. doi: 10.1371/journal.pcbi.1006705 (PMC6370239; doi:10.1371/journal.pcbi.1006705)
Supplement: S2 Fig — Distribution of χ1 angles observed in unified binding site residues. Values were normalized on a per-family basis before radar binning such that each unique protein sequence is represented equally, regardless of family size. Data for: A) All unified binding-site residues, B) Arg, C) Asn, D) Asp, E) Cys, F) Gln, G) Glu, H) His, I) Ile, J) Leu, K) Lys, L) Met, M) Phe, N) Ser, O) Thr, P) Trp, Q) Tyr, R) Val. (DOCX) [file pcbi.1006705.s002.docx]

**
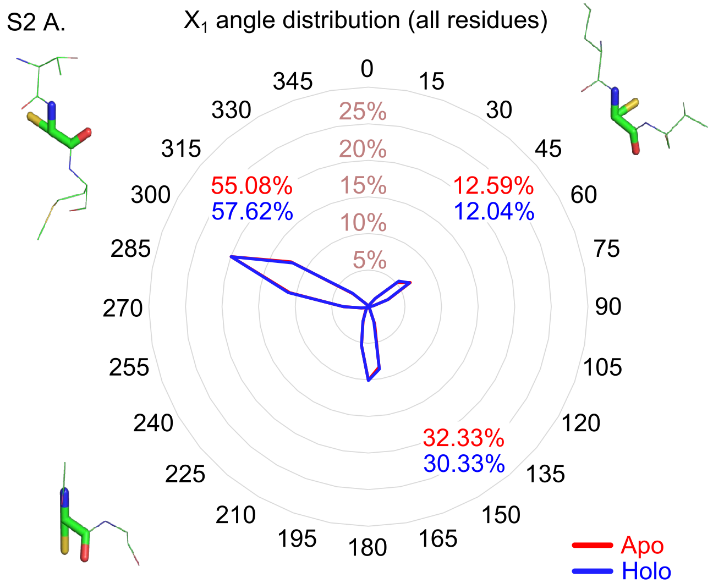

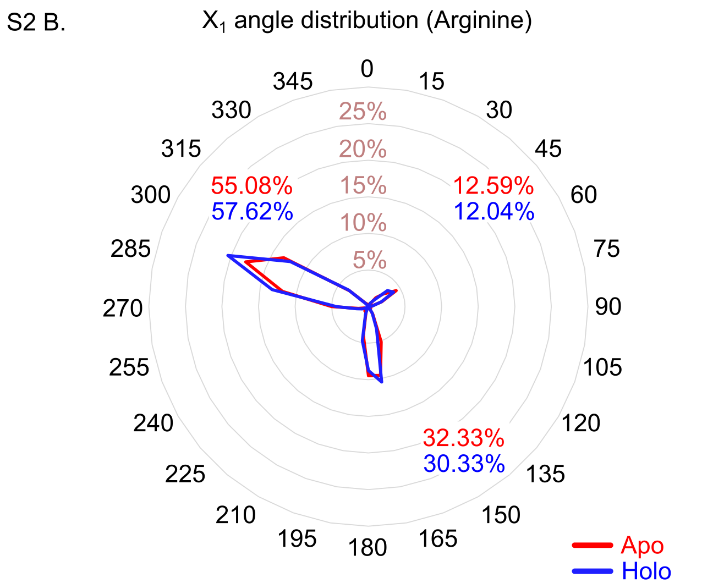

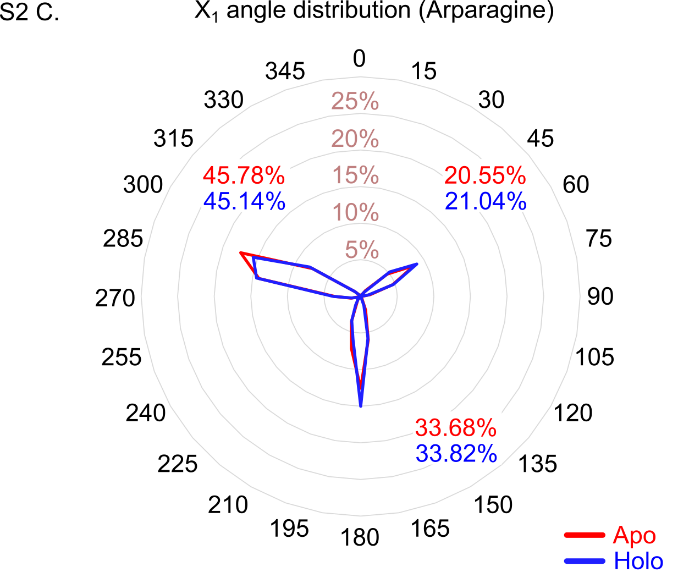

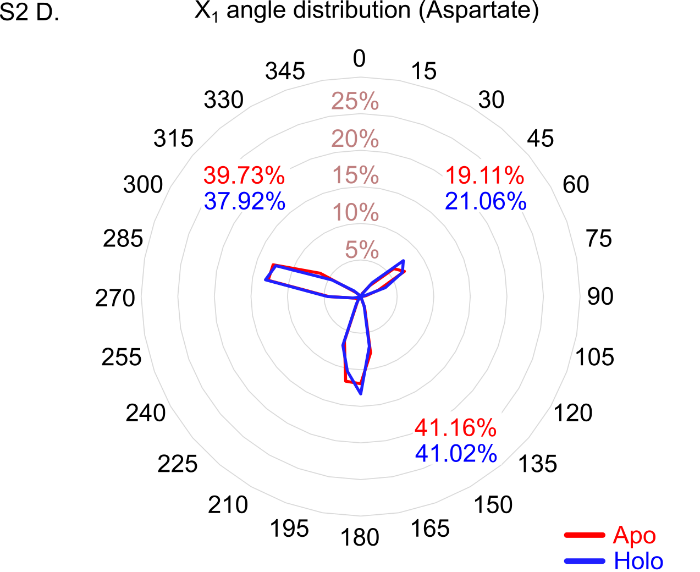

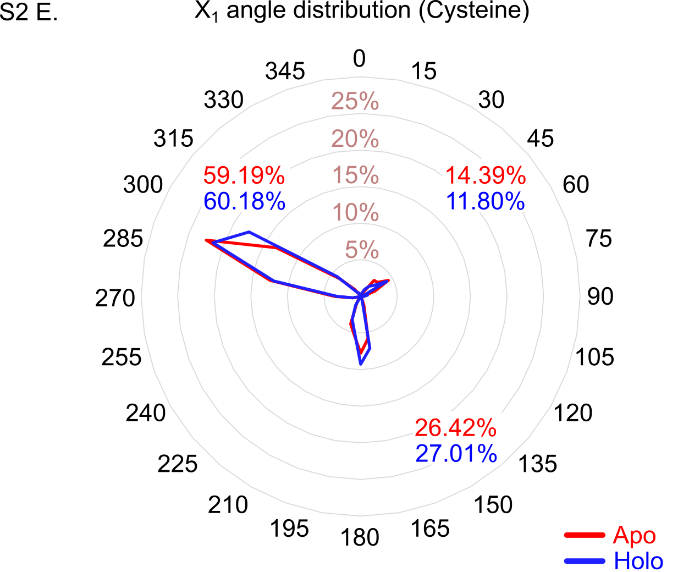

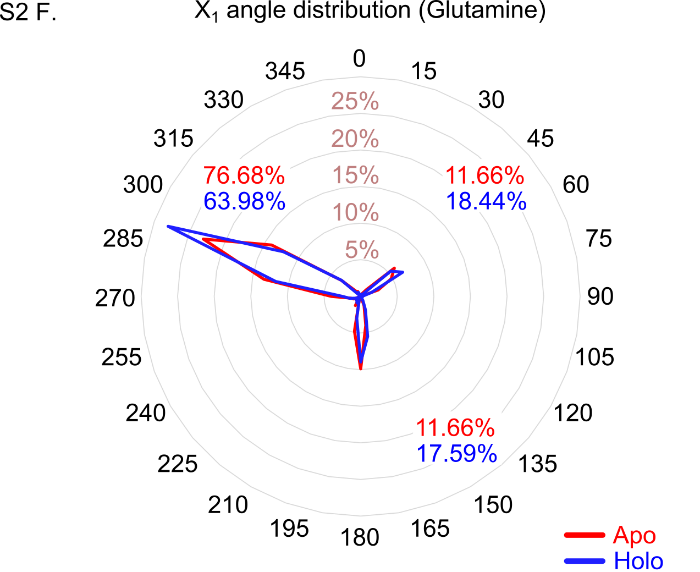

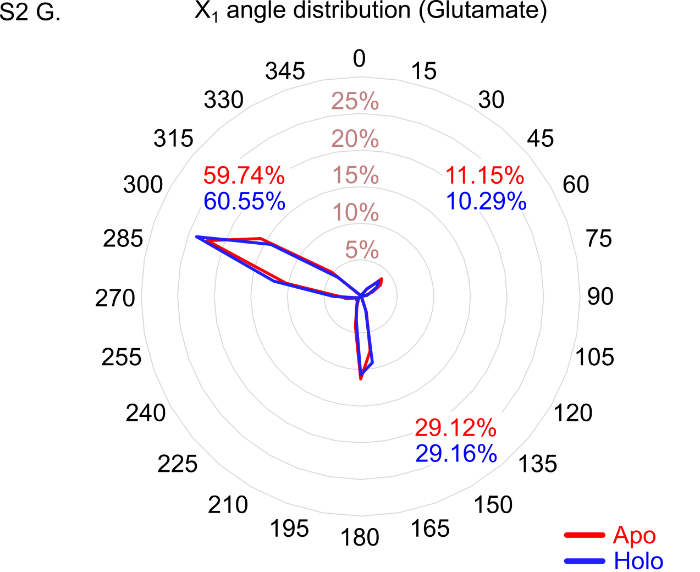

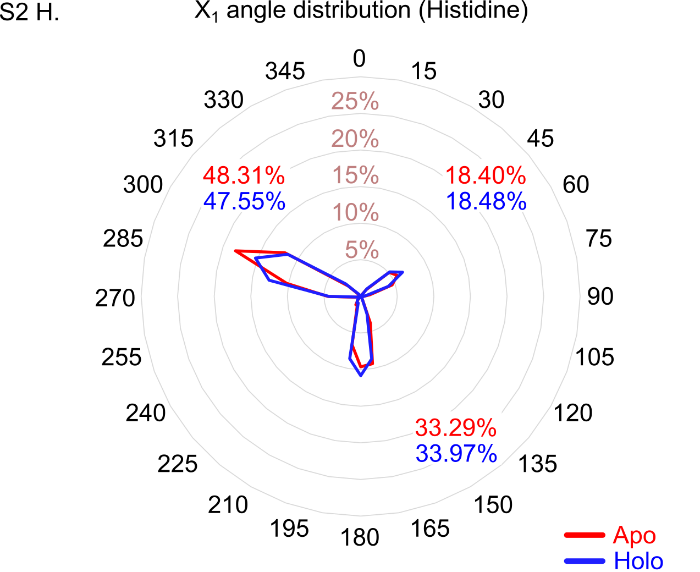

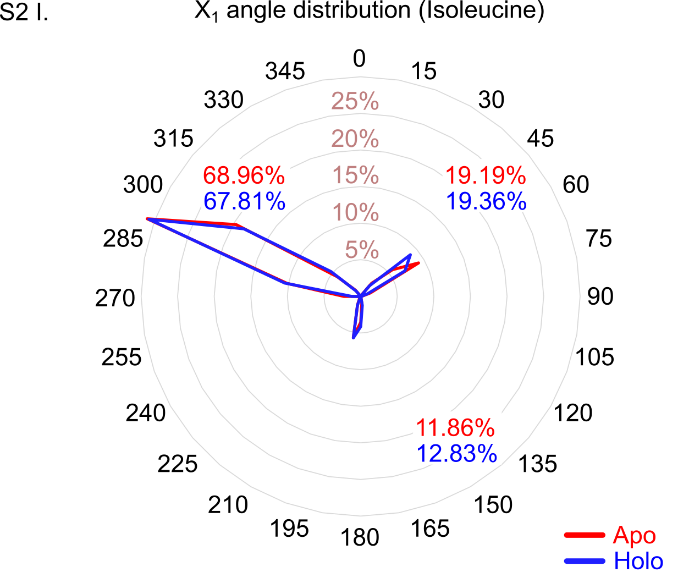

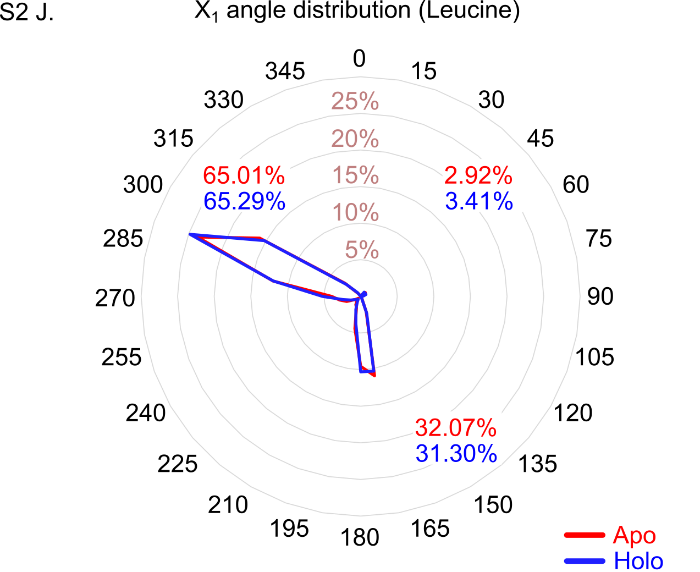

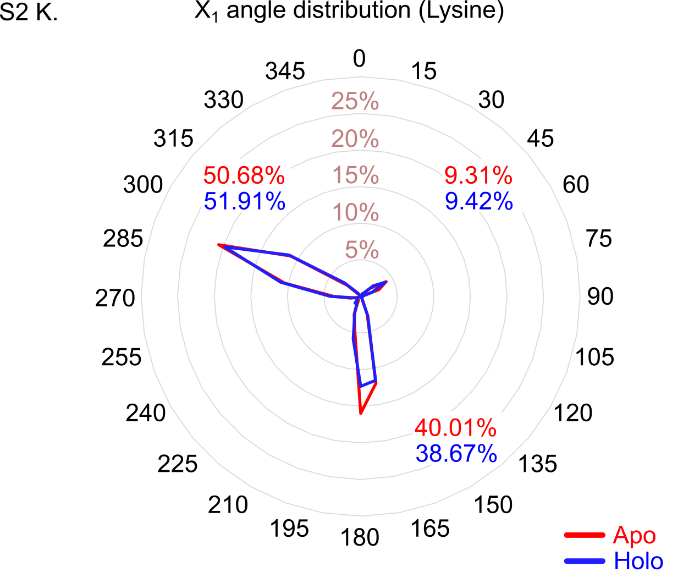

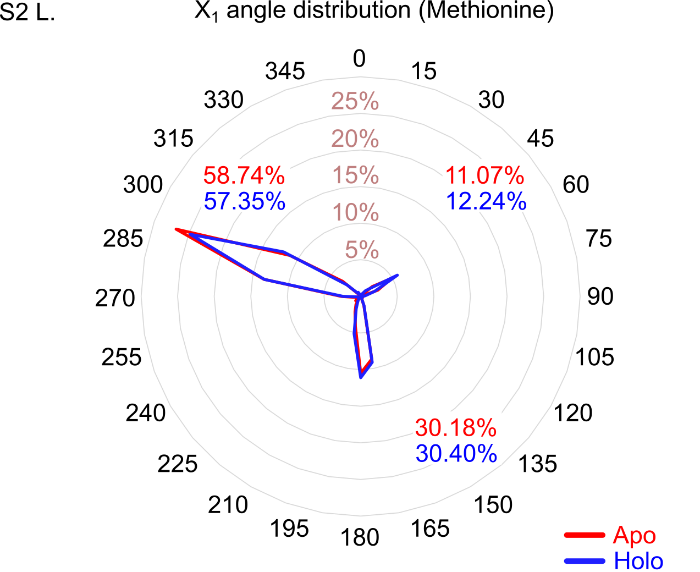

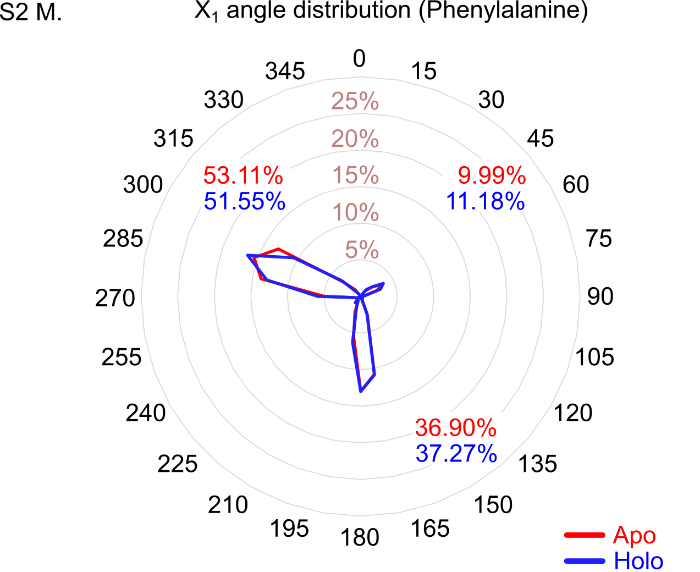

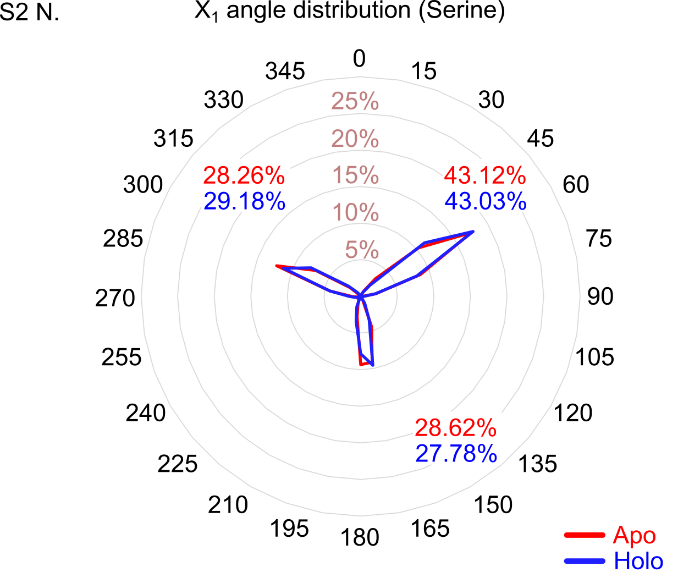

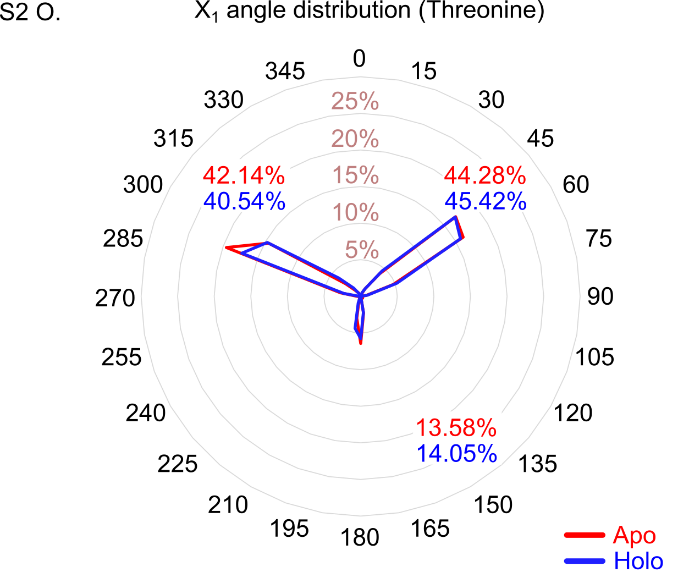

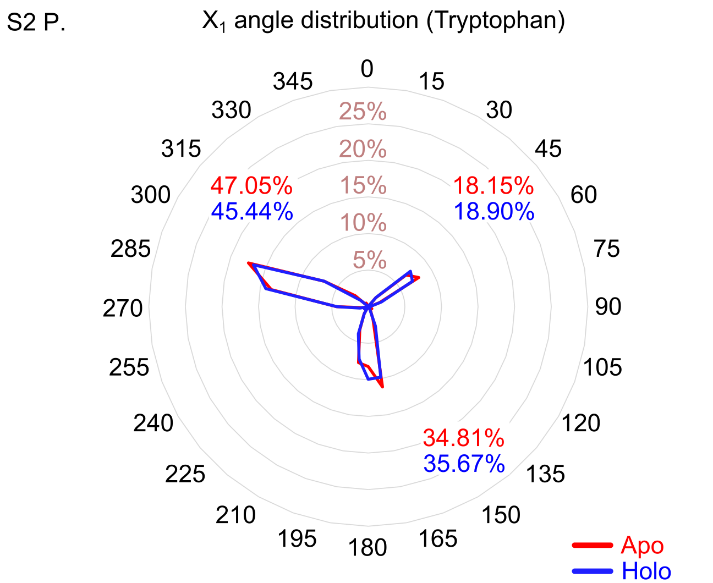

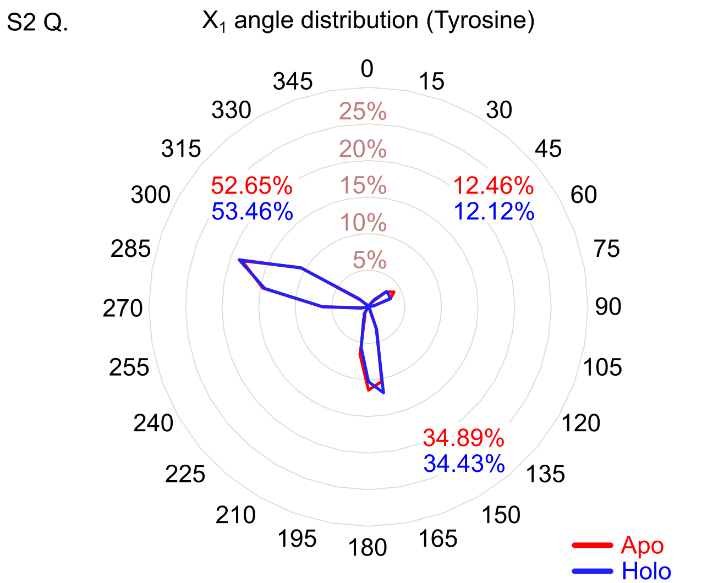

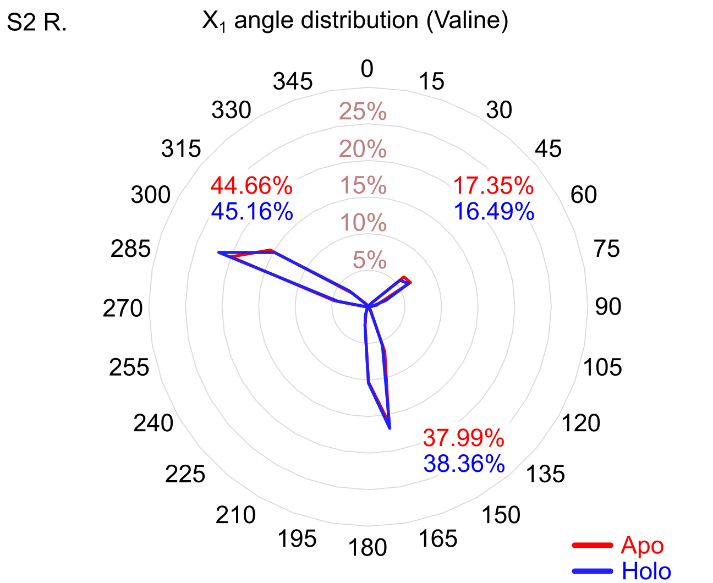
**

**Figure_S2. Radar plots of χ_1_ angle distributions.** Distribution of χ_1_ angles observed in unified binding site residues. Values were normalized on a per-family basis before radar binning such that each unique protein sequence is represented equally, regardless of family size. Data for: A) All UBS residues, B) Arg, C) Asn, D) Asp, E) Cys, F) Gln, G) Glu, H) His, I) Ile, J) Leu, K) Lys, L) Met, M) Phe, N) Ser, O) Thr, P) Trp, Q) Tyr, R) Val.
